# Chalybeates in Dropsy

**Published:** 1835

**Authors:** 


### VII.—Chalybeates in Dropsy.

Dr. William Pettit, of Columbiana county, Ohio, has sent us a paper commending the use of Rubigo Ferri mixed with port wine, in the treatment of Dropsy. He thinks that the influence of Dr. Rush, in favor of the antiphlogistic method, has had, in many instances, an unfavorable effect on the practice of American physicians. Dr. P. states, that his attention was some time since directed to this medicine, by Dr. Isaac Parker, of Jefferson county, in this state, an old and respectable physician, who assured him that he was generally successful in the management of ascites and anasarca, by means of the following preparation:

℞ Carbonate of Iron ℥i.

Port Wine Hj.

Mix.

Agitate when used, and give half an ounce three or four times a day.

Dr. Pettit has given two successful cases in detail. We shall abridge one of them.

A lady aged 36 years, after the birth of her third child, became ascitic, and in despite of a great variety of means, administered by different persons, both in and out of the profession, continued in that state for several months. When called to attend her, Dr. P. resorted to tapping and drew off thirty-eight pints of fluid. He then gave her the German mixture of cream of tartar, sulphate of potash, squills and tartar emetic, but symptoms of reaccumulation ensued. In this exigency he had resource to the rubigo ferri in port wine, which was perfectly successful. She subsequently became pregnant, when dropsical symptoms again returned, but were promptly arrested by a resort to the chalybeate.

We think it probable, that while some cases of dropsy requiring antiphlogistics are improperly treated in the opposite way, others are prolonged, or rendered fatal, by an adherence to the former, when the latter method is required. The books would no doubt furnish authority for giving sedative and refrigerant diuretics, like those administered to this lady, after the operation of paracentesis, but such a practice rests on no solid foundation. Tapping should never be employed as long as there is active or even subacute peritonitis, and after it is subdued, whether the water be drawn off or not, tonics, among which none are superior to the chalybeate preparations, are undoubtedly the proper remedies.

We would here inquire whether any of our readers have given a trial, in dropsy, to the sulphate of iron, in drachm doses, as recommended by the physicians of the Italian school? If so, we should like to be informed of the results of their practice, and would make them public.

